# The *Anisakis* Transcriptome Provides a Resource for Fundamental and Applied Studies on Allergy-Causing Parasites

**DOI:** 10.1371/journal.pntd.0004845

**Published:** 2016-07-29

**Authors:** Fiona J. Baird, Xiaopei Su, Ibukun Aibinu, Matthew J. Nolan, Hiromu Sugiyama, Domenico Otranto, Andreas L. Lopata, Cinzia Cantacessi

**Affiliations:** 1 Centre for Biodiscovery & Molecular Development of Therapeutics, James Cook University, Townsville, Australia; 2 Australian Institute of Tropical Health and Medicine, James Cook University, Townsville, Australia; 3 Department of Veterinary Medicine, University of Cambridge, Cambridge, United Kingdom; 4 School of Applied Sciences, RMIT University, Bundoora, Australia; 5 Department of Pathology and Pathogen Biology, Royal Veterinary College, University of London, Hatfield, United Kingdom; 6 Department of Parasitology, National Institute of Infectious Diseases, Tokyo, Japan; 7 Department of Veterinary Medicine, University of Bari, Valenzano, Italy; Universidade Federal de Minas Gerais, BRAZIL

## Abstract

**Background:**

Food-borne nematodes of the genus *Anisakis* are responsible for a wide range of illnesses (= anisakiasis), from self-limiting gastrointestinal forms to severe systemic allergic reactions, which are often misdiagnosed and under-reported. In order to enhance and refine current diagnostic tools for anisakiasis, knowledge of the whole spectrum of parasite molecules transcribed and expressed by this parasite, including those acting as potential allergens, is necessary.

**Methodology/Principal Findings:**

In this study, we employ high-throughput (Illumina) sequencing and bioinformatics to characterise the transcriptomes of two *Anisakis* species, *A*. *simplex* and *A*. *pegreffii*, and utilize this resource to compile lists of potential allergens from these parasites. A total of ~65,000,000 reads were generated from cDNA libraries for each species, and assembled into ~34,000 transcripts (= Unigenes); ~18,000 peptides were predicted from each cDNA library and classified based on homology searches, protein motifs and gene ontology and biological pathway mapping. Using comparative analyses with sequence data available in public databases, 36 (*A*. *simplex*) and 29 (*A*. *pegreffii*) putative allergens were identified, including sequences encoding ‘novel’ *Anisakis* allergenic proteins (i.e. cyclophilins and ABA-1 domain containing proteins).

**Conclusions/Significance:**

This study represents a first step towards providing the research community with a curated dataset to use as a molecular resource for future investigations of the biology of *Anisakis*, including molecules putatively acting as allergens, using functional genomics, proteomics and immunological tools. Ultimately, an improved knowledge of the biological functions of these molecules in the parasite, as well as of their immunogenic properties, will assist the development of comprehensive, reliable and robust diagnostic tools.

## Author Summary

Nematodes within the genus *Anisakis* (i.e. *A*. *simplex* and *A*. *pegreffii*, also known as herring worms) are the causative agents of the fish-borne gastrointestinal illness known as ‘anisakiasis’, with infections resulting in symptoms ranging from mild gastric forms to severe allergic reactions leading to urticaria, gastrointestinal and/or respiratory signs and/or anaphylaxis (‘allergic anisakiasis’). Despite significant advances in knowledge of the pathobiology of allergic anisakiasis, thus far, the exact number and nature of parasite molecules acting as potential allergens are currently unknown; filling this gap is necessary to the development of robust and reliable diagnostics for allergic anisakiasis which, in turn, underpins the implementation of effective therapeutic strategies. Here, we use RNA-Seq and bioinformatics to sequence and annotate the transcriptomes of *A*. *simplex* and *A*. *pegreffii*, and, as an example application of these resources, mine this data to identify and characterise putative novel parasite allergens based on comparisons with known allergen sequence data from other parasites and other organisms.

## Introduction

Foodborne diseases include a range of illnesses transmitted *via* the ingestion of foodstuffs contaminated with a variety of chemical compounds and pathogenic microorganisms, including parasites [1–3]. Whilst the global disease burden and costs linked to these illnesses are difficult to estimate, it has been calculated that foodborne infections have cost the Australian economy alone $1.249 billion in 2006 [4]. Amongst the parasites responsible for foodborne diseases, nematodes within the genus *Anisakis* (i.e. *A*. *simplex* and *A*. *pegreffii*, also known as herring worms) are the causative agents of the fish-borne gastrointestinal illness ‘anisakiasis’. Since the 1960s, over 20,000 cases have been reported worldwide [5]; however, this number is most likely severely underestimated. Indeed, in Japan, where the consumption of raw or undercooked fish is common, ~2000–3000 new cases of anisakiasis occur each year [5].

The life cycle of *Anisakis* is indirect, with cetaceans such as dolphins and whales harbouring the dioecious adult nematodes in their gastrointestinal tract; female *Anisakis* release unembryonated eggs that are excreted in the aquatic environment *via* the faeces. Following development into first-, second- and third-stage larvae (L1s, L2s and L3s, respectively), these hatch from the eggs and are ingested by crustacean hosts. When infected crustaceans are ingested by suitable paratenic hosts, such as fish or squid, L3s penetrate the intestine and encapsulate in tissues, particularly those of the liver and the mesentery. Following ingestion of L3-containing paratenic hosts by a suitable cetacean host, L3s develop to fourth-stage larvae (L4s) and subsequently to adult males and females [5]. Humans are accidental hosts for *Anisakis*, with the infection occurring *via* the ingestion of L3-containing raw or undercooked fish. In humans, the infection is usually self-limiting, and common symptoms range from epigastric pain, nausea, vomiting and low-grade fever (= gastric form), to intermittent or constant abdominal pain with possible complications such as peritonitis and/or ascites (= intestinal form). ‘Ectopic’ anisakiasis occurs when the ingested larvae penetrate the gut wall and undergo a somatic migration to other viscera [5]. However, individuals infected by *Anisakis* spp. can also become sensitised to parasite allergens, leading to the onset of allergic anisakiasis (the most significant form of disease), with symptoms ranging from urticaria, gastrointestinal and/or respiratory signs and/or anaphylaxis [5, 6]. Other than the ingestion of nematodes, allergic reactions can also be elicited by accidental exposure to hidden antigens in processed (including cooked) fish and fish products [7, 8], as well as inhalation of *Anisakis* allergens [9, 10].

To date, a total of 14 *A*. *simplex* allergens have been identified [see ref. 5]. Most of these allergens have been detected in the parasite excretory/secretory (ES) products, with Ani s 1, Ani s 5 and Ani s 7 being recognised by serum antibodies in the majority of individuals affected by allergic anisakiasis [11, 12]. A recent proteomic investigation of L3s of *A*. *simplex* led to the identification of 17 novel putative allergens, which included structural proteins (e.g. myosin-4), as well as a number of enzymes (e.g. one enolase and one endochitinase) [13]. These antigens, derived from both ES and somatic components (the latter released following death and disintegration of the larvae), act as triggers for the activation of complex immunological and cellular host defences, which often result in allergic sensitisation [14]. However, thus far, the exact number and nature of parasite molecules acting as potential allergens are currently unknown. Next-generation sequencing and bioinformatics now provide rapid and cost-effective opportunities to investigate the fundamental biology of parasites of medical significance [see 15], and to build curated molecular databases for in-depth analyses of specific sets of parasite genes and gene products [16, 17] of biological and/or medical relevance. For instance, the study of the transcriptome (= the complete set of mRNAs transcribed by a cell, tissue or organism at any one time) represents a powerful approach to identify and characterise thousands of parasite transcripts simultaneously, which can then be screened for one or more sequences of interest. In this study, we sequence and characterise the transcriptomes of *A*. *simplex* and *A*. *pegreffii* in order to build annotated datasets for fundamental studies of the biology of these parasites. Using these resources, we compile lists of putative parasite allergens based on comparative analyses with known allergen sequence data available in public databases.

## Materials and Methods

### Parasite material

L3s of *A*. *pegreffii* (AP) and *A*. *simplex* sensu stricto (AS) were collected between July and October 2013 in Tokyo, Japan. Whole fish (*Scomber japonicas–*chub mackerel; *Scomber australasicus–*blue mackerel and *Trachurus japonicus*–Japanese jack mackerel) were purchased from retail outlets and fish markets; the viscera were removed and inspected for anisakid L3 parasites and, when detected, these were collected and washed three times in saline solution. For each parasite, a segment of the caudal end was sectioned for molecular species identification [18], while the remaining portions were individually stored at -80°C for subsequent RNA extraction. For molecular identification, a region spanning the internal transcribed spacer 1 (ITS1), the 5.8S and the internal transcribed spacer 2 (ITS2) of the ribosomal DNA (rDNA) was amplified using the primer pairs 5’- GTCGAATTCGTAGGTGAACCTGCGGAAGGATCA-3’ and reverse: 3’- GCCGGATCCGAATCCTGGTTAGTTTCTTTTCCT-5’ and the thermocycling protocol described by D’Amelio et al. [18]. PCR amplicons were digested using the restriction enzyme *Hinf*I for identification of the diagnostic molecular fingerprints for *A*. *simplex* s.s. (~620, 250 and 100 bp) and *A*. *pegreffii* (~370, 200 and 250 bp), and *Hha*I for differentiation between *A*. *simplex* s.s. (~550 and 430 bp) and *A*. *berlandi* (formerly *A*. *simplex* C) (~550, 300 and 130 bp), respectively [18].

### RNA isolation and Illumina sequencing

RNA was extracted from three samples of each AS (100 L3s per sample) and AP (100 L3s per sample) using the Trizol reagent (Invitrogen, Life Technologies, Carlsbad, USA), and DNAse-treated using Turbo DNA-*free* (Ambion, Austin, USA) according to the manufacturer’s instructions. The amounts and integrity of total RNA were determined using a 2100 BioAnalyzer (Agilent Technologies, Santa Clara, California, USA). Polyadenylated (PolyA+) RNA was purified from 10 mg of total RNA from each AS and AP using Sera-Mag oligo(dT) beads, fragmented to a length of 100–500 nucleotides and reverse transcribed to cDNA using random hexamers. The size-fractionated cDNA was end-repaired and adaptor-ligated according to the manufacturer’s protocol (Illumina). Ligated products of 200 bp were excised from agarose gels and PCR-amplified (15 cycles) [cf. 19]. Products were cleaned using a MinElute PCR purification kit (Qiagen, Hilden, Germany) and paired-end sequenced on an Illumina HiSeq 2000 [20] according to the manufacturer’s protocol.

### Sequence trimming and assembly

Following removal of adapter sequences and sequences with suboptimal read quality (i.e., PHRED score of 32.0) using the filter_fq script (https://github.com/greatfireball/filter_fq), the remaining 100-bp paired-read reads generated from the cDNA libraries from AS and AP were each assembled *de novo* using the program Trinity, which combines three independent software modules, i.e. Inchworm, Chrysalis, and Butterfly [21] (http://trinityrnaseq.sourceforge.net/). Briefly, a representative set of transcripts was assembled, including full-length transcripts of dominant isoforms and unique portions of alternatively spliced transcripts (Inchworm); next, portions of alternatively spliced transcripts and/or unique portions of paralogous genes were clustered and a de Brujin graph was constructed for each cluster of transcripts (Chrysalis); finally, de Brujin graphs were analysed simultaneously and full-length transcripts for alternatively spliced isoforms derived from paralogous genes were reported (Butterfly) [21]. In order to further reduce redundancy and generate comprehensive transcriptome datasets for each AS and AP, the resulting sequences, designated ‘Unigenes’, were compared across biological replicates of the same species, and ‘Clusters’ of Unigenes were generated based on sequence similarity (70% similarity cut-off).

### Annotation

The non-redundant, assembled datasets were then compared with transcriptomic and protein sequence data available for *Anisakis* spp. in the EST database of NCBI (http://www.ncbi.nlm.nih.gov/nucest/?term=anisakis) and in the WormBase ParaSite database (http://parasite.wormbase.org/Anisakis_simplex_prjeb496/Info/Index/) (e-value cut-off: 1e-05), and annotated using an established approach [cf. 19, 22]. Briefly, assembled Clusters and Unigenes (= contigs) were compared using the BLASTn and BLASTx algorithms to sequences available in WormBase (www.wormbase.org), in the nucleotide sequence collection (Nt) of NCBI (www.ncbi.nlm.nih.gov), and in the non-redundant (Nr) (www.ncbi.nlm.nih.gov), SwissProt (http://expasy.org/), Kyoto Encyclopedia of Genes and Genomes (KEGG; http://www.genome.jp/kegg/) and Clusters of Orthologous Groups (COG; http://www.ncbi.nlm.nih.gov/COG/) databases, respectively (e-value cut-off < 0.00001). Putative homologues in other representatives of Clade III nematodes (i.e. *A*. *suum* and *Toxocara canis*; [23, 24]), other nematodes and organisms other than nematodes were also identified (e-value cut-off: 1e-05). Gene Ontology terms (GO, http://www.geneontology.org/) [25] were assigned to computationally translated AS and AP transcripts based on similarity to peptide sequences in the SwissProt database, according to the categories ‘Biological Process’, ‘Cellular Component’ and ‘Molecular Function’, using the Blast2GO software using default settings [26].

### Identification of putative allergens

In order to compile a list of putative allergens from AS and AP, predicted peptides from each of these species were compared with sequence data currently available in the AllergenOnline database (http://www.allergenonline.com/; January 2015 release) using the BLASTp algorithm (e-value cut-off: 1e-05; 70% identity match). At the time of the analyses (December 2015), this database contained ~1,900 peer-reviewed “protein sequence entries categorised into 744 taxonomic protein groups of unique proven or putative allergens (food, airway, venom/salivary and contact)” compiled from the GenBank, RefSeq and TPA nucleotide sequence repositories, as well as from the SwissProt, PIR, PRF and PDB protein sequence databases (http://www.allergenonline.com/). Computationally translated amino acid sequences from AS and AP were also compared, by BLASTp, with the sets of putative allergens described by Arcos et al. [27] and Fæste et al. [13] (e-value cut-off: 1e-05).

## Results

### The *Anisakis* transcriptomes

Paired-end Illumina sequencing of AS and AP cDNA libraries resulted in a total number of 64,065,430 and 65,508,456 raw reads, respectively (Table 1). Raw reads generated in the present study have been deposited in the Sequence Read Archive (SRA) database of NCBI (http://www.ncbi.nlm.nih.gov/sra) under study number SRP070744. Following pre-processing of raw reads, assembly and grouping of assembled Unigenes into sequence Clusters, a total of 34,746 (AS) and 33,747 (AP) transcripts were obtained. Conceptual translation of AS and AP transcripts resulted in 18,842 and 17,732 full-length predicted proteins, respectively (Table 1), of which 71.5% (AS) and 71.7% (AP) could be annotated *via* BLAST searches against the Nr, SwissProt, KEGG, COG and/or the GO databases (Table 1). Overall, 96.7%, 47.5% and 33.5% of AS transcripts and 88.0%, 90.5% and 73.8% of predicted proteins matched known nucleotide and amino acid sequences from *A*. *simplex* (http://parasite.wormbase.org/Anisakis_simplex_prjeb496/Info/Index/), as well as the phylogenetically related ascarid nematodes *A*. *suum* and *T*. *canis*, respectively; for AP, 96.6%, 46.1% and 33.2% of transcripts matched *A*. *simplex*, *A*. *suum* and *T*. *canis* sequences, respectively, while comparisons of amino acid sequence data resulted in 88.0%, 89.8% and 75.0% of AP predicted peptides, respectively, matching available peptide sequences from these two nematodes (Table 1).

**Table 1:** Transcriptome sequence data for third-stage larvae of *Anisakis simplex* and *Anisakis pegreffii* prior to and following assembly, and nucleotide and predicted peptide sequence data annotation (Nr = non-redundant; KEGG = Kyoto Encyclopedia of Genes and Genomes; COG = Clusters of Orthologous Groups of Proteins; GO = Gene Ontology).

	*Anisakis simplex*	*Anisakis pegreffii*
Sequencing output		
No. of raw reads	64,065,430	65,508,456
No. of clean reads	59,110,560	60,362,154
Average length	100	100
GC content (%)	43.40	43.16
Transcript Assembly		
Unigenes^a^		
Total no.	62,967	61,258
Mean Length ± SD (nt)	444 ± 692	465 ± 732
N50^b^	1195	1304
Contigs^a^		
No. of clusters	12,277	11,394
No. of singletons (Unigenes)	22,469	22,353
Total no.	34,746	33,747
Mean Length ± SD (nt)	1173 ± 1105	1190 ± 1123
N50^b^	2027	2082
Transcript coverage (%)^c^		
100–90	32,902	31,893
90–80	1,620	1,583
80–70	189	229
70–60	27	29
60–50	2	2
50–40	6	1
No. of contigs matching *Anisakis simplex* genes (WormBase ParaSite)	33,610	32,592
*Ascaris suum* genes	16,500	15,569
*Toxocara canis* genes	11,625	11,222
Predicted peptides		
No. of predicted peptides by BLAST	12,914	12,195
ESTScan	5,928	5,537
Total	18,842	17,732
No. of predicted peptides matching *Anisakis simplex* proteins (WormBase ParaSite)	16,571	15,668
*Ascaris suum* proteins	17,045	15,949
*Toxocara canis* proteins	13,907	13,298
Annotation		
No. of predicted peptides with matches in Nr	12,699	12,022
Swissprot	7,747	7,563
KEGG (no. of biological pathways)	8,945 (126)	8,629 (124)
COG	7,677	7,387
GO	5,561	5,357
Total no. of annotated predicted peptides	13,472	12,705

^a^Assembled transcripts prior to (= Unigenes) and following sequence clustering (= Contigs) (see Materials and Methods)

^b^Length N (in bp) for which 50% of all nucleotides sequenced are assembled in Contigs of length ≤ N

^c^Percentage of a transcript covered by reads. The value is calculated as a ratio of the number of bases in a transcript covered by unique mapping reads to the number of total bases in that transcript.

A total of 5,561 (29.5%) and 5,357 (30.2%) AS and AP predicted proteins, respectively, could be assigned GO terms, while 8,945 (AS, 47.5%) and 8,629 (AP, 48.7%) matched homologous proteins in the KEGG database associated to 126 (AS) and 124 (AP) distinct biological pathways (Table 1). The 7,677 (AS, 40.7%) and 7,387 (AP, 41.7%) predicted proteins with matches in the COG database could be assigned to at least one of 25 functional categories, of which ‘general function prediction’ (AS = 15.7%; AP = 16.0%), ‘replication, recombination and repair’ (AS = 7.8%; AP = 7.7%), and ‘transcription’ (AS = 7.2%; AP = 7.4%), were the most represented (S1 Fig).

### Putative novel *Anisakis* allergens

Comparative analyses of AS and AP predicted peptides with sequence data available in the AllergenOnline Database (http://www.allergenonline.com/) resulted in a total number of 38 (AS) and 31 (AP) matches, respectively, including 22 (AS) and 18 (AP) sequences matching previously known *Anisakis* allergens (S1 Table). Of the 16 (AS) and 13 (AP) remaining predicted peptides, 10 (AS) and 8 (AP) also matched protein sequences described as putative novel allergens by Arcos et al. [28] and Faeste et al. [13] (Table 2). BLAST comparisons between sets of putative allergens identified in AS and AP revealed six sequences unique to AS, which included a heat shock 70 kDa protein (ID: Unigene9825_AS1A), two fructose-bisphosphate aldolases A, a 60S ribosomal protein and two putative allergens matching sequences previously characterised in *A*. *suum* and *Ascaris lumbricoides* (Table 2); one sequence encoding a heat shock 70 kDa protein (ID: Unigene16173_AP1A) was unique to AP (Table 2). All of the putative novel allergens identified matched homologous allergenic proteins in arthropods (AS = 7; AP = 9), fungi (AS = 6; AP = 6), plants (AS = 3; AP = 3), other helminths (AS = 3; AP = 1) and fish (AS = 3; AP = 1) (Table 2). Of the putative allergens characterised in the present study, cyclophilins (AS and AP) and two predicted proteins of unknown function (AS) were identified in *Anisakis* for the first time (Table 2). Summaries of GO annotation and KEGG pathway analysis information for the whole transcriptomes of AS and AP are shown in S1 Fig, whilst the complete lists of AS and AP assembled transcripts, together with corresponding predicted peptides and annotation information, are given in S2 Table.

**Table 2:** Putative novel allergens identified in the transcriptomes from third-stage larvae of *Anisakis simplex* and *Anisakis pegreffii*, based on homology of predicted amino acid sequences with known allergens in the AllergenOnline database (http://www.allergenonline.com/about.shtml). (e-value cut-off: <1e-05, identity cut-off: >70%). (Nr = non-redundant database; GO = Gene Ontology; KEGG = Kyoto Encyclopedia of Genes and Genomes; BP = Biological Process; CC = Cellular Component; MF = Molecular Function).

Unigene ID	Size (bp)	Uniquely mapped reads	Closest match in Nr	GO annotation	KEGG pathway annotation	Closest match in AllergenOnline database	IUIS nomenclature^a^	Taxonomy^b^
*Anisakis simplex*								
CL2584.Contig1_AS1A*	644	335597	Heat shock 70 kDa protein [*Anisakis pegreffii*]	Cell, Cell part, Membrane (CC)	Spliceosome, Protein processing in endoplasmic reticulum, Endocytosis	Heat shock 70 kDa protein	Unassigned	*Pci*, *Dfa*, *Che*
Unigene14515_AS1A*	438	11166	Heat shock 70kDa protein 8 [*Danio rerio*]	Response to stimulus (BP)	Spliceosome, Protein processing in endoplasmic reticulum, Endocytosis	Heat shock 70 kDa protein	Unassigned	*Pci*, *Dfa*, *Che*
Unigene14555_AS1A*	213	75345	Heat shock cognate 71 kDa protein [*Danio rerio*]	Response to stimulus (BP)	Spliceosome, Protein processing in endoplasmic reticulum, Endocytosis	Heat shock 70 kDa protein	Unassigned	*Pci*
Unigene7175_AS1A*	276	21823	Heat shock protein 70 b2 [*Ascaris suum*]	Cell, Cell part, Membrane (CC)	Spliceosome, Protein processing in endoplasmic reticulum, Endocytosis	Heat shock 70 kDa protein	Unassigned	*Pci*, *Dfa*, *Che*
Unigene9825_AS1A*	258	16179	Heat shock 70 kDa protein c [*Ascaris suum*]	Cellular process, Growth, Multi-organism process, Reproduction, Response to stimulus, Reproductive process, Single-organism process (BP); Cell, Cell part, Membrane, Membrane-enclosed lumen, Organelle part (CC)	Protein processing in endoplasmic reticulum, Protein export	Heat shock 70 kDa protein	Unassigned	*Pci*, *Dfa*, *Che*
Unigene10469_AS1A	171	48012	Peptidyl-prolyl cis-trans isomerase 3 [*Ascaris suum*]	Cellular component organization or biogenesis, Growth, Rhythmic process, Signaling (BP); Extracellular region, Macromolecular complex, Membrane, Organelle (CC)	None	Cyclophilin	Unassigned	*Dca*, *Bpe*, *Dfa*
Unigene11979_AS1A	159	1631	Peptidyl-prolyl cis-trans isomerase e [*Ascaris suum*]	None	None	Cyclophilin	Unassigned	*Dfa*
Unigene15335_AS1A	134	50809	Ppia protein, partial [*Danio rerio*]	Membrane (CC)	None	Cyclophilin	Unassigned	*Dfa*, *Cro*, *Bpe*, *Dca*, *Msy*, *Afu*
Unigene6668_AS1A	172	18480	Peptidyl-prolyl cis-trans isomerase 3 [*Toxocara canis*]	Extracellular region, Membrane (CC)	None	Cyclophilin	Unassigned	*Dca*, *Bpe*, *Dfa*, *Cro*
Unigene2527_AS1A**	347	61681	Fructose-bisphosphate aldolase 2 [*Ascaris suum*]	Metabolic process (BP); Organelle part (CC)	Metabolic pathways, Biosynthesis of secondary metabolites, Glycolysis/Gluconeogenesis, Fructose and mannose metabolism, Pentose phosphate pathway, Carbon fixation in photosynthetic organisms	Fructose-bisphosphate aldolase A	Thu a 3.0101	*Tal*
Unigene8467_AS1A**	351	24201	Fructose-bisphosphate aldolase 1 [*Ascaris suum*]	Cellular process, Metabolic process (BP)	Metabolic pathways, Biosynthesis of secondary metabolites, Glycolysis/Gluconeogenesis, Fructose and mannose metabolism, Pentose phosphate pathway, Carbon fixation in photosynthetic organisms	Fructose-bisphosphate aldolase A	Thu a 3.0101	*Tal*
Unigene17381_AS1A*	68	174503	Ribosomal protein L3 [*Danio rerio*]	Cell, Cell part, Macromolecular complex, Membrane, Membrane-enclosed lumen, Organelle, Organelle part (CC); Structural molecule activity (MF)	Ribosome	60S ribosomal protein L3	Unassigned	*Afu*
Unigene10513_AS1A*	431	133980	Enolase [*Anisakis simplex*]	Cellular component organization or biogenesis, Cellular process, Metabolic process (BP); Extracellular region, Macromolecular complex, Membrane (CC)	Metabolic pathways, Biosynthesis of secondary metabolites, RNA degradation, Glycolysis/Gluconeogenesis	Enolase 3–2	Sal s 2.0101	*Ssa*
Unigene7252_AS1A**	203	15510	Sigma class glutathione S-transferase [*Baylisascaris schroederi*]	None	Metabolic pathways, mRNA surveillance pathway, Glutathione metabolism, Arachidonic acid metabolism	Glutathione S-transferase 1	Asc s 13.0101	*Asu*
CL1712.Contig1_AS1A	294	8338	Allergen, partial [*Ascaris suum*]	None	None	ABA-1 allergen, partial	Unassigned	*Alu*, *Asu*
CL1712.Contig2_AS1A	160	2996	Polyprotein allergen/antigen, partial [*Ascaris suum*]	None	None	ABA-1 allergen, partial	Unassigned	*Alu*, *Asu*
*Anisakis pegreffii*								
Unigene13566_AP1A*	176	94213	Heat shock cognate 71 kDa protein [*Danio rerio*]	Response to stimulus (BP)	Spliceosome, Protein processing in endoplasmic reticulum, Endocytosis	Heat shock 70 kDa protein	Unassigned	*Pci*
Unigene13814_AP1A*	438	2533	Heat shock 70kDa protein 8 [*Danio rerio*]	Response to stimulus (BP)	Spliceosome, Protein processing in endoplasmic reticulum, Endocytosis	Heat shock 70 kDa protein	Unassigned	*Pci*, *Dfa*, *Che*
Unigene16173_AP1A*	88	22129	Inducible heat shock protein 70 [*Tigriopus californicus*]	Response to stimulus (BP); Organelle (CC)	Spliceosome, Protein processing in endoplasmic reticulum, Endocytosis, Protein export	Heat shock 70 kDa protein	Unassigned	*Pci*, *Dfa*, *Che*
Unigene16415_AP1A*	138	38735	Heat shock protein 70 b2 [*Ascaris suum]*	Cell, Cell part, Membrane (CC)	Spliceosome, Protein processing in endoplasmic reticulum, Endocytosis	Heat shock 70 kDa protein	Unassigned	*Dfa*, *Che*
CL872.Contig1_AP1A*	644	367002	Heat shock protein 70 [*Anisakis pegreffii*]	Cell, Cell part, Membrane (CC)	Spliceosome, Protein processing in endoplasmic reticulum, Endocytosis	Heat shock 70 kDa protein	Unassigned	*Pci*, *Dfa*, *Che*
Unigene21614_AP1A*	81	9769	Heat shock protein 70, partial [*Neobathyscia mancinii*]	Response to stimulus (BP)	Spliceosome, Protein processing in endoplasmic reticulum, Endocytosis	Heat shock 70 kDa protein	Unassigned	*Pci*, *Dfa*, *Che*
CL1265.Contig1_AP1A	171	64890	Peptidyl-prolyl cis-trans isomerase 3 [*Ascaris suum*]	Extracellular region, Membrane (CC)	None	Cyclophilin	Unassigned	*Dca*, *Bpe*, *Dfa*, *Cro*
CL1927.Contig1_AP1A	159	1062	Peptidyl-prolyl cis-trans isomerase e [*Ascaris suum*]	None	None	Cyclophilin	Unassigned	*Dfa*
CL1927.Contig2_AP1A	159	3029	Peptidyl-prolyl cis-trans isomerase e [*Ascaris suum*]	None	None	Cyclophilin	Unassigned	*Dfa*
Unigene16460_AP1A	145	3867	Ppia protein, partial [*Danio rerio*]	Membrane (CC)	None	Cyclophilin	Unassigned	*Dca*, *Bpe*, *Dfa*, *Cro*
Unigene10435_AP1A	172	28438	Peptidyl-prolyl cis-trans isomerase 3 [*Toxocara canis*]	Extracellular region, Membrane (CC)	None	Cyclophilin	Unassigned	*Dca*, *Bpe*, *Dfa*, *Cro*
Unigene2939_AP1A*	431	93319	Enolase [*Anisakis simplex*]	Cellular process, Metabolic process (BP); Extracellular region, Macromolecular complex, Membrane (CC)	Metabolic pathways, Biosynthesis of secondary metabolites, RNA degradation, Glycolysis/Gluconeogenesis	Enolase 3–2	Sal s 2.0101	*Ssa*
Unigene6873_AP1A**	203	21192	Sigma class glutathione S-transferase [*Baylisascaris schroederi*]	None	Metabolic pathways, mRNA surveillance pathway, Glutathione metabolism, Arachidonic acid metabolism	Glutathione S-transferase 1	Asc s 13.0101	*Asu*

^a^Systematic allergen nomenclature approved by the World Health Organization and International Union of Immunological Societies (WHO/IUIS) Allergen Nomenclature Sub-committee

^b^Abbreviations of homologous species of matched allergens (alphabetical): *Ascaris lumbricoides (Alu)*, *Ascaris suum (Asu)*, *Aspergillus fumigatus (Afu)*, *Betula pendula (Bpe)*, *Blattella germanica (Bge)*, *Catharanthus roseus (Cro)*, *Cladosporium herbarum (Che)*, *Daucus carota (Dca)*, *Dermatophagoides farina (Dfa)*, *Malassezia sympodialis (Msy)*, *Penicillium citrinum (Pci)*, *Salmo salar (Ssa)*, *Thunnus albacares (Tal)*, *Tyrophagus putrescentiae (Tpu)*; Taxonomy: Nematodes: *Alu*, *Asu*; Fungi: *Afu*, *Che*, *Msy*, *Pci*; Plant: *Bpe*, *Cro*, *Dca*; Arthropods: *Bge*, *Dfa*, *Tpu*; Fish: *Ssa*, *Tal*.

*Also identified by Arcos et al. [28] and Fæste et al. [13].

**Also identified by Fæste et al. [13].

## Discussion

In this study, we characterise the transcriptomes of two *Anisakis* species, and provide the scientific community with a resource to explore the biology of these parasites, as well as their allergenic properties, using proteomics and immunological tools. The caudal extremities of the larvae used in this study were removed in order to unequivocally confirm species identification by PCR-coupled RFLP of the of ITS1-5.8S-ITS2 region of the rDNA, thus potentially leading to biases in the sets of *Anisakis* transcripts identified and characterised. Nevertheless, we consider these datasets to represent a comprehensive snapshot of the complements of genes transcribed by the L3s of these parasites. Indeed, prior to this study, only 913 *Anisakis* transcripts were present in the EST database of NCBI (http://www.ncbi.nlm.nih.gov/nucest/?term=anisakis); however, a draft genome sequence for this parasite, accompanied by large-scale transcriptomic sequence data to support gene predictions, are currently available from the WormBase ParaSite database as part of the ‘50 Helminth Genome Initiative’ (http://www.sanger.ac.uk/science/collaboration/50hgp). Though, given that these resources are as yet unpublished, we opted to assemble the short reads generated in this study *de novo*, and compare the resulting full-length cDNAs (contigs) and, subsequently, the amino acid sequences predicted from these, to *Anisakis* transcripts and predicted proteins (respectively) on the WormBase ParaSite database. Overall, 96.7% and 96.6% of the transcripts assembled in the present study matched *Anisakis* nucleotide sequence data in the latter database, thus providing support to the reliability and robustness of our assembly (cf. Table 1). Conversely, BLAST comparisons of *Anisakis* nucleotide and predicted amino acid sequence data generated in this study with available transcripts and protein sets from other ascarids [23, 24] revealed higher sequence similarities between *Anisakis* and *A*. *suum* than between the former and *T*. *canis* (see Table 1). Currently, the relationships amongst Clade III nematodes (also referred to as suborder Spirurina; [28]) are defined according to phylogenetic analyses of the small subunit of the ribosomal RNA (SSU rRNA) [29, 30]. In one of these investigations, SSU rRNA sequences from *Ascaris* spp. and *Toxocara* group together to the exclusion of the *Anisakis* spp. counterparts [30], likely reflecting the similarities in morphology and fundamental biology between the former (‘terrestrial’) species. While the elucidation of the systematic and phylogenetic relationships between *Anisakis*, *Ascaris* and *Toxocara* species are beyond the scope of the present work, the availability of large-scale transcriptomic and genomic datasets for all of these parasites (http://www.sanger.ac.uk/science/collaboration/50hgp; [23, 24]) represents a useful resource for future, comprehensive phylogenomic studies of ascarid nematodes.

In this study, we aimed to sequence and annotate the transcriptomes of two *Anisakis* species, in order to build a molecular resource from which to draw information on the set(s) of molecules responsible for evoking allergic responses in the human host. Two recently published key studies [13, 27] utilised sera from human patients with IgE against *A*. *simplex* and positive to the skin prick test, coupled with mass spectrometry-based analyses of reactive *Anisakis* proteins to identify and characterise putative novel nematode allergens. Both studies revealed a broad array of potentially allergenic *Anisakis* molecules, with individual sera binding to multiple protein bands, which resulted in a substantial inter-individual variability of binding patterns [13]. This finding suggests that the whole complement of *Anisakis* allergens is yet to be fully defined, and supports the application of NGS technologies and bioinformatics to assist in this quest. While all of the putative novel allergens identified by Arcos et al. [27] and Fæste et al. [13] matched AS and AP amino acid sequences inferred from cDNAs generated in this study, only subsets of these also matched known allergens in the AllergenOnline database (cf. Table 2). The most likely explanation for this observation is technical and is related to the inevitable incompleteness of the AllergenOnline database which, while regularly updated and manually curated, relies on the collection of sequence data submitted by end-users to public sequence repositories and designated as ‘allerg*’, or extracted directly from peer-reviewed publications (www.allergenonline.com). On the other hand, the absence of some previously identified putative *Anisakis* allergens from the AllergenOnline database is justified by the fact that the allergenic properties of these molecules are yet to be fully elucidated [13, 27].

Amongst the putative allergens characterised in the present study, sequences encoding heat shock proteins 70 (HSPs 70) and enolases were identified in both AS and AP datasets, and had been previously detected using immune-proteomic approaches [13, 27]. Based on direct comparisons with sequence data in the Nr database of NCBI, these (and other) sequences displayed high sequence similarity with HSPs from *Danio rerio* (cf. Table 2). While this finding is likely to be linked to the overrepresentation of sequences from zebrafish in Nr compared with sequence data from parasitic nematodes, contamination of *Anisakis* mRNA with that from the fish host cannot be excluded. HSPs are a family of highly conserved proteins that play primary roles in maintaining cellular homeostasis but whose increased expression in the presence of conditions of stress such as sudden changes in temperature, injuries and infections, is responsible for the activation of a cascade of immune-molecular events that culminate in inflammatory responses [31]. In particular, in a recent study [32], the expression of an HSP 70 from *A*. *pegreffii* was increased in L4s compared with L3s, which may be linked to a response of the parasite to the heat stress that immediately follows infection [32]. HSPs 70 from mites, black flies, midges and cockroaches are known allergens and mediators of allergic contact hypersensitivity [33, 34]; in addition, levels of HSPs 70 are increased in the sputum and plasma of asthmatic patients compared with healthy controls [35], while antibodies against these proteins are associated with a number of immunological disorders, such as allergy to metals [36]. Based on this knowledge, it is therefore plausible that antibodies against *Anisakis* HSPs 70, whose expression is increased upon host infection [32], may play a key role in allergic responses to these parasites. Enolases are also recognised as major allergenic proteins in plants, fish, fungi, cockroaches and biting insects [37]. In a previous study, anti-enolase antibodies were detected in sera from mice experimentally infected with *A*. *simplex* L3s or exposed to parasite crude protein extracts, but not from mouse sera raised against the parasite excretory-secretory antigens [38]; in the same study, anti-enolase antibodies could not be detected in the sera of human patients infected with *A*. *simplex*, which led the authors to speculate that these molecules do not offer a sufficient antigenic stimulus to act as allergens [38]. Conversely, IgE against *Anisakis* enolases were detected in *Anisakis*-allergic patients by Fæste et al. [13], thus supporting the role of these molecules in allergic anisakiasis. However, it is worthwhile to note that, while sensitisation to *Anisakis* allergens usually follows the ingestion of fish and/or fish products, these products can also contain very similar allergens to those from the parasites ([e.g. 39]; cf. Table 2). Importantly, recent studies [39] have demonstrated the role of fish beta-enolase and fructose-bisphosphate aldolase as allergenic stimuli in patients sensitised to cod, salmon and tuna, thus further complicating the diagnosis of ‘true’ sensitisation to *Anisakis* allergens. In addition, tropomyosin (Ani s 3), one of the most immunogenic proteins known to man, was also first discovered in *Anisakis* and subsequently identified in over 150 invertebrate species (including shellfish and mites) [see ref. 1]. This muscle protein, which contains a coiled-coiled α-helical structure, is responsible for exacerbated immune responses in over 30% of the world’s population, and may be involved in the known cases of cross-allergenicity between mites and *Anisakis* [40].

Of the molecules inferred as novel putative allergens, sequences encoding peptidyl-prolyl cis-trans isomerases (cyclophilins) were identified in both AS and AP (cf. Table 2). Cyclophilins belong to a family of conserved proteins present in both prokaryotes and eukaryotes, and thought to play key roles in a range of human inflammatory diseases, including rheumatoid arthritis and asthma [41]. Human cyclophilins also act as self-antigens, being recognised by serum IgE from individuals sensitised to environmental cyclophilins, such as those in pollens [see 42]. Cyclophilins have also been identified in a range of parasitic nematode species (e.g. *Angiostrongylus cantonensis*, *Dirofilaria immitis* and *Haemonchus contortus*; [43–45]) and are thought to operate as catalysts and chaperones in cuticle synthesis [46]; in particular, cyclophilins were identified in the excretory-secretory products of *H*. *contortus* and recognised by sheep hyper-immune sera [47]. Interestingly, cyclophilins were also detected by immunological screening of a cDNA library from the zoonotic cestode *Echinococcus granulosus*, causing cystic echinococcosis (CE), with sera from infected human subjects that had displayed allergic (skin) reactions [48]; sera from these patients did not recognise the homologous human cyclophilins, nor that from the yeast *Malassezia furfur*, thus supporting the hypothesis that the parasite cyclophilin was responsible for the allergic reactions observed in patients with CE [48]. Based on this information, we hypothesise a role of cyclophilins in the array of molecules responsible for allergic anisakiasis, a hypothesis that requires testing.

Amongst the novel putative allergens in AS were two sequences with high sequence similarity to ABA-1 proteins, members of the nematode polyprotein allergens (NPAs) from *A*. *suum* (cf. Table 2). These proteins are synthetised as repetitive polyproteins, which are subsequently cleaved during post-translational processing into multiple functional units with fatty-acid binding properties [49]. NPAs from *Ascaris* and other parasitic nematodes (e.g. *Brugia malayi*) are associated with hypersensivity responses in infected individuals [see 50]. In addition, no IgE cross-reactivity could be detected between human sera (from asthmatic patients from an area where ascariasis is endemic and exposure to mites is common) probed with recombinant *Ascaris* ABA-1 and mite fatty-acid binding proteins (FABPs), thus suggesting that parasites are solely responsible for allergic reactions against this protein [51]. Given the high level of sequence similarity (at both nucleotide and protein levels) between *Anisakis* sequences encoding for ABA-1 proteins identified in this study and the *Ascaris* counterparts, it is plausible that these molecules also contribute to the onset of allergic anisakiasis.

While our work represents a step forward in the application of NGS technologies towards building a molecular infrastructure for research on allergic anisakiasis, gaps still exist in our knowledge of the complex host-parasite relationships which culminate in the exacerbated immune reactions observed in individuals infected by *Anisakis*. The completion, curation and refinement of the whole genome sequence of *A*. *simplex* (http://www.sanger.ac.uk/science/collaboration/50hgp) will assist filling these gaps, by providing a solid resource for fundamental functional explorations of these relationships. Ultimately, the discovery of the whole array of parasite molecules responsible for immune hypersensitivity in *Anisakis* (and other parasites) will set the basis of future studies aimed at developing comprehensive, reliable and robust diagnostic tools, which will assist clinicians in choosing appropriate intervention strategies and effectively assessing their outcomes.

## Supporting Information

S1 Fig Functional annotation of the *Anisakis* transcriptomes. Summary of functional annotation information linked to predicted peptides inferred from the transcriptomes of *Anisakis simplex* and *Anisakis pegreffii* third stage larvae as inferred *via* comparisons with sequence data available in the Kyoto Encyclopedia of Genes and Genomes (KEGG, Level 1; expressed as percentage of Unigenes mapping to conserved biological pathways) (**A**), Gene Ontology (GO; Level 2), according to the categories ‘Biological Process’, ‘Cellular Component’ and ‘Molecular Function’ (**B**) and Clusters of Orthologous Groups of Proteins (COG) (**C**) databases. (**D**) Venn diagram illustrating the number of assembled transcripts shared between *A*. *simplex* and *A*. *pegreffii*, and of transcripts unique to each species (e-value cut-off: 1e-15).
(PDF)

S1 Table Predicted peptides with homology to previously known *Anisakis* allergens. Predicted peptides inferred from the transcriptomes of third stage larvae of *Anisakis simplex* and *Anisakis pegreffii* with homology to previously known *Anisakis* allergens (e-value cut-off: <1e-5, identity cut-off: >70%) available in the AllergenOnline database (http://www.allergenonline.com/about.shtml) (Nr = non-redundant database).
(DOCX)

S2 Table Lists of assembled transcripts from *Anisakis simplex* and *Anisakis pegreffii*. Complete lists of assembled transcripts and predicted peptides inferred from the transcriptomes of third stage larvae of *A*. *simplex* and *A*. *pegreffii*, and corresponding nucleotide and predicted amino acid sequences and functional annotation.
(XLSX)
