# Supplementary figures and images for: The Anisakis Transcriptome Provides a Resource for Fundamental and Applied Studies on Allergy-Causing Parasites
Source: PLoS Negl Trop Dis. 2016 Jul 29;10(7):e0004845. doi: 10.1371/journal.pntd.0004845 (PMC4966942; doi:10.1371/journal.pntd.0004845)

(A)

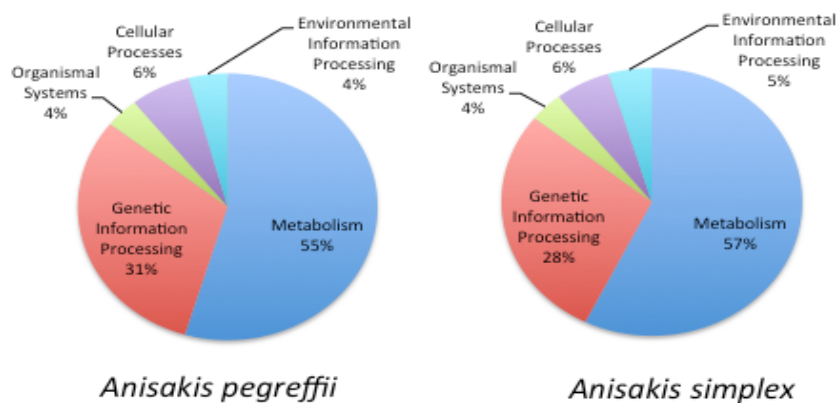

(B)

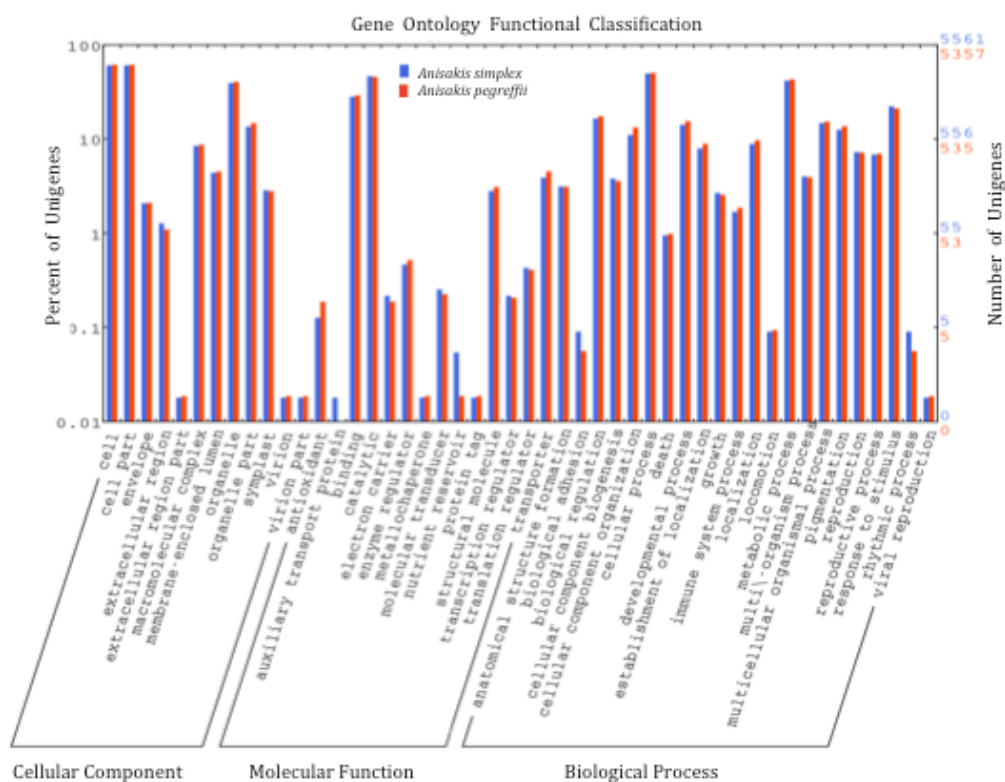

(C)

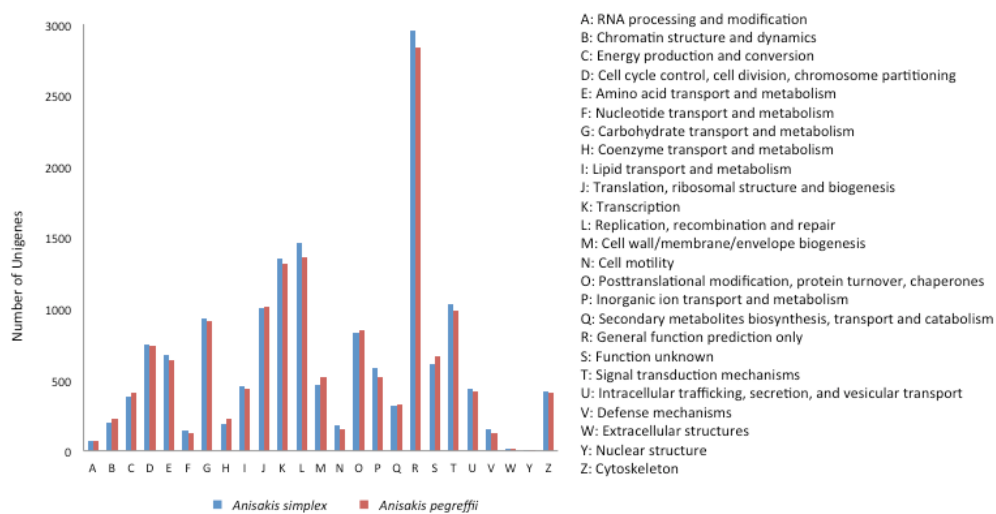

**(D)**

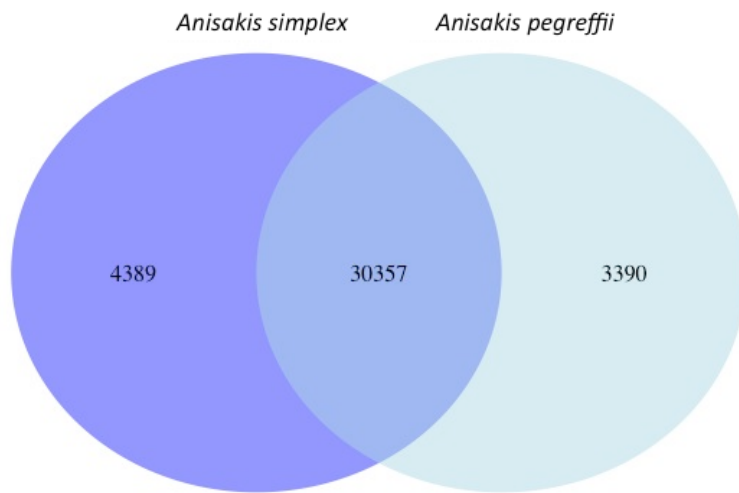

Supplement: S1 Fig — Summary of functional annotation information linked to predicted peptides inferred from the transcriptomes of Anisakis simplex and Anisakis pegreffii third stage larvae as inferred via comparisons with sequence data available in the Kyoto Encyclopedia of Genes and Genomes (KEGG, Level 1; expressed as percentage of Unigenes mapping to conserved biological pathways) (A), Gene Ontology (GO; Level 2), according to the categories ‘Biological Process’, ‘Cellular Component’ and ‘Molecular Function’ (B) and Clusters of Orthologous Groups of Proteins (COG) (C) databases. (D) Venn diagram illustrating the number of assembled transcripts shared between A. simplex and A. pegreffii, and of transcripts unique to each species (e-value cut-off: 1e-15). (PDF) [file pntd.0004845.s001.pdf]
