# Supplementary material for: The Anisakis Transcriptome Provides a Resource for Fundamental and Applied Studies on Allergy-Causing Parasites
Source: PLoS Negl Trop Dis. 2016 Jul 29;10(7):e0004845. doi: 10.1371/journal.pntd.0004845 (PMC4966942; doi:10.1371/journal.pntd.0004845)
Supplement: S1 Table — Predicted peptides inferred from the transcriptomes of third stage larvae of Anisakis simplex and Anisakis pegreffii with homology to previously known Anisakis allergens (e-value cut-off: <1e-5, identity cut-off: >70%) available in the AllergenOnline database (http://www.allergenonline.com/about.shtml) (Nr = non-redundant database). (DOCX) [file pntd.0004845.s002.docx]

**Supplementary Table 1. Predicted peptides with homology to previously known *Anisakis* allergens**. Predicted peptides inferred from the transcriptomes of third stage larvae of *Anisakis simplex* and *Anisakis pegreffii* with homology to previously known *Anisakis* allergens (e-value cut-off: <1e-5, identity cut-off: >70%) available in the AllergenOnline database. (<http://www.allergenonline.com/about.shtml>) (Nr = non-redundant database).

| Unigene ID | Size (bp) | Closest match in Nr | Closest match in AllergenOnline |
| --- | --- | --- | --- |
| *Anisakis simplex* |  |  |  |
| CL1474.Contig1_AS1A | 369 | Paramyosin [*Toxocara canis*] | Paramyosin, Ani s 2 |
| CL1474.Contig2_AS1A | 468 | Paramyosin; Allergen=Ani s 2 [*Anisakis simplex*] | Paramyosin, Ani s 2 |
| CL1474.Contig3_AS1A | 240 | Paramyosin; Allergen=Ani s 2 [*Anisakis simplex*] | Paramyosin, Ani s 2 |
| Unigene6540_AS1A | 58 | paramyosin isoform [*Anisakis simplex*] | Paramyosin |
| Unigene9109_AS1A | 84 | Paramyosin; Allergen=Ani s 2 [*Anisakis simplex*] | Paramyosin |
| CL2300.Contig3_AS1A | 281 | Ani s 12 allergen precursor [*Anisakis simplex*] | Ani s 12 allergen |
| CL2300.Contig4_AS1A | 256 | Ani s 12 allergen [*Anisakis simplex*] | Ani s 12 allergen and precursor |
| Unigene8429_AS1A | 193 | Major allergen Ani s 1 [*Anisakis simplex*] | Ani s 1 allergen |
| Unigene12957_AS1A | 94 | Ani s 4 allergen [*Anisakis simplex*] | Ani s 4 allergen |
| CL2474.Contig1_AS1A | 156 | Ani s 9 allergen precursor [*Anisakis simplex*] | Ani s 9 allergen precursor |
| CL2802.Contig2_AS1A | 130 | Ani s 11-like protein precursor [*Anisakis simplex*] | Ani s 11 allergen precursor |
| CL2802.Contig3_AS1A | 124 | Ani s 11-like protein precursor [*Anisakis simplex*] | Ani s 11 allergen precursor |
| CL3720.Contig2_AS1A | 142 | Ani s 11-like protein precursor [*Anisakis simplex*] | Ani s 11 allergen precursor |
| Unigene9538_AS1A | 98 | Ani s 11 allergen precursor [*Anisakis simplex*] | Ani s 11 allergen precursor |
| CL61.Contig1_AS1A | 193 | SXP/RAL-2 family protein 2 isoform 1 [*Anisakis simplex*] | SXP/RAL-2 family protein 2 |
| Unigene11210_AS1A | 162 | SXP/RAL-2 family protein [*Anisakis simplex*] | SXP/RAL-2 family protein |
| Unigene11525_AS1A | 1424 | UA3-recognized allergen, partial [*Anisakis simplex*] | UA3-recognized allergen, partial |
| Unigene13306_AS1A | 64 | Troponin-like protein [*Anisakis simplex*] | Troponin-like protein |
| Unigene12103_AS1A | 276 | Tropomyosin [*Ascaris lumbricoides*] | Tropomyosin |
| Unigene8904_AS1A | 63 | Troponin isoform 2 [*Ascaris suum*] | Troponin C |
| CL140.Contig1_AS1A | 653 | Ani s 13 [*Anisakis simplex*] | Ani s 13 allergen |
| Unigene6130_AS1A | 1269 | Ani s 14 [*Anisakis simplex*] | Ani s 14 allergen |
| *Anisakis pegreffii* |  |  |  |
| Unigene3736_AP1A | 369 | Paramyosin [*Toxocara canis*] | Paramyosin |
| Unigene9538_AP1A | 214 | Paramyosin; Allergen=Ani s 2 [*Anisakis simplex*] | Paramyosin |
| Unigene9537_AP1A | 84 | Paramyosin; Allergen=Ani s 2 [*Anisakis simplex*] | Paramyosin |
| Unigene8558_AP1A | 174 | Major allergen Ani s 1 | Ani s 1 allergen |
| CL1482.Contig1_AP1A | 94 | Ani s 4 allergen [*Anisakis simplex*] | Ani s 4 allergen |
| CL1482.Contig2_AP1A | 94 | Ani s 4 allergen [*Anisakis simplex*] | Ani s 4 allergen |
| Unigene10343_AP1A | 139 | Ani s 9 allergen precursor [*Anisakis simplex*] | Ani s 9 allergen precursor |
| Unigene725_AP1A | 101 | Ani s 10 allergen precursor [*Anisakis simplex*] | Ani s 10 allergen precursor |
| Unigene8184_AP1A | 169 | SXP/RAL-2 family protein [*Anisakis simplex*] | SXP/RAL-2 family protein |
| CL1065.Contig1_AP1A | 1288 | UA3-recognized allergen, partial [*Anisakis simplex*] | UA3-recognized allergen, partial |
| CL1065.Contig2_AP1A | 1380 | UA3-recognized allergen, partial [*Anisakis simplex*] | UA3-recognized allergen, partial |
| Unigene2933_AP1A | 64 | Troponin-like protein [*Anisakis simplex*] | Troponin-like protein |
| Unigene9089_AP1A | 63 | Troponin isoform 2 [*Ascaris suum*] | Troponin C |
| Unigene11858_AP1A | 276 | Tropomyosin [*Ascaris lumbricoides*] | Tropomyosin |
| Unigene11859_AP1A | 276 | Tropomyosin [*Ascaris lumbricoides*] | Tropomyosin |
| CL2325.Contig1_AP1A | 442 | Ani s 13 [*Anisakis simplex*] | Ani s 13 allergen |
| Unigene5124_AP1A | 448 | Ani s 14 [*Anisakis simplex*] | Ani s 14 allergen |
